# Supplementary material for: Wild dogs at stake: deforestation threatens the only Amazon endemic canid, the short-eared dog (Atelocynus microtis)
Source: R Soc Open Sci. 2020 Apr 22;7(4):190717. doi: 10.1098/rsos.190717 (PMC7211836; doi:10.1098/rsos.190717)
Supplement: Additional information [file rsos190717supp2.docx]

**Wild dogs at stake: Deforestation threatens the only Amazon endemic canid, the short-eared dog (*Atelocynus microtis*)**

Rocha et al.

**SUPPORTING INFORMATION**

Table S1. Selected variables expected to influence short-eared dog (Atelocynus microtis) distribution. Variables used in the modeling phase are highlighted in bold.

| **Environmental variables** | **Resolution** | **Date** |
| --- | --- | --- |
| **Climatic^a^** |  |  |
| **BIO01. Annual Mean Temperature** | 30 seconds | 1970-2000^d^ |
| **BIO02. Mean Diurnal Temperature Range** | 30 seconds | 1970-2000^d^ |
| **BIO03. Isothermality** | 30 seconds | 1970-2000^d^ |
| BIO04. Temperature Seasonality | 30 seconds | 1970-2000^d^ |
| BIO05. Max Temperature of Warmest Month | 30 seconds | 1970-2000^d^ |
| BIO06. Min Temperature of Coldest Month | 30 seconds | 1970-2000^d^ |
| BIO07. Temperature Annual Range | 30 seconds | 1970-2000^d^ |
| BIO08. Mean Temperature of Wettest Quarter | 30 seconds | 1970-2000^d^ |
| BIO09. Mean Temperature of Driest Quarter | 30 seconds | 1970-2000^d^ |
| BIO10. Mean Temperature of Warmest Quarter | 30 seconds | 1970-2000^d^ |
| BIO11. Mean Temperature of Coldest Quarter | 30 seconds | 1970-2000^d^ |
| **BIO12. Annual Precipitation** | 30 seconds | 1970-2000^d^ |
| BIO13. Precipitation of Wettest Month | 30 seconds | 1970-2000^d^ |
| BIO14. Precipitation of Driest Month | 30 seconds | 1970-2000^d^ |
| **BIO15. Precipitation Seasonality** | 30 seconds | 1970-2000^d^ |
| BIO16. Precipitation of Wettest Quarter | 30 seconds | 1970-2000^d^ |
| BIO17. Precipitation of Driest Quarter | 30 seconds | 1970-2000^d^ |
| **BIO18. Precipitation of Warmest Quarter** | 30 seconds | 1970-2000^d^ |
| BIO19. Precipitation of Coldest Quarter | 30 seconds | 1970-2000^d^ |
| **Topographic^b^** |  |  |
| Digital Elevation Above Sea Level (from SRTM) | 3 arc-seconds | 1996 |
| **Land cover^c^** |  |  |
| **ESA GlobCover Project 2009** | 10 arc-seconds | 2009 |
| ^a^ www.wordclim.org |  |  |
| ^b^ https://lta.cr.usgs.gov/SRTM1Arc |  |  |
| ^c^ http://due.esrin.esa.int/page_globcover.php |  |  |
| ^d^ average for the years 1970-2000 |  |  |

Table S2. Code used to reclassify GlobCover layer to make it comparable with simulated future forest cover layers of the Amazon basin developed by Soares-Filho et al. (2006).

| **Values** | **Label** | **New values** | **New Label** |
| --- | --- | --- | --- |
| 14 | Rainfed croplands | 1 | Deforested |
| 20 | Mosaic cropland (50-70%) / vegetation (grassland/shrubland/forest) (20-50%) | 1 | Deforested |
| 30 | Mosaic vegetation (grassland/shrubland/forest) (50-70%) / cropland (20-50%) | 1 | Deforested |
| 40 | Closed to open (>15%) broadleaved evergreen or semi-deciduous forest (>5m) | 2 | Forest |
| 50 | Closed (>40%) broadleaved deciduous forest (>5m) | 2 | Forest |
| 60 | Open (15-40%) broadleaved deciduous forest/woodland (>5m) | 2 | Forest |
| 110 | Mosaic forest or shrubland (50-70%) / grassland (20-50%) | 2 | Forest |
| 120 | Mosaic grassland (50-70%) / forest or shrubland (20-50%) | 2 | Forest |
| 130 | Closed to open (>15%) (broadleaved or needleleaved, evergreen or deciduous) shrubland (<5m) | 2 | Forest |
| 140 | Closed to open (>15%) herbaceous vegetation (grassland, savannas or lichens/mosses) | 2 | Forest |
| 150 | Sparse (<15%) vegetation | 3 | No forest |
| 160 | Closed to open (>15%) broadleaved forest regularly flooded (semi-permanently or temporarily) - Fresh or brackish water | 2 | Forest |
| 170 | Closed (>40%) broadleaved forest or shrubland permanently flooded - Saline or brackish water | 2 | Forest |
| 180 | Closed to open (>15%) grassland or woody vegetation on regularly flooded or waterlogged soil - Fresh, brackish or saline water | 2 | Forest |
| 190 | Artificial surfaces and associated areas (Urban areas >50%) | 3 | No forest |
| 200 | Bare areas | 1 | Deforested |
| 210 | Water bodies | 3 | No Forest |
| 220 | Permanent snow and ice | 3 | No Forest |


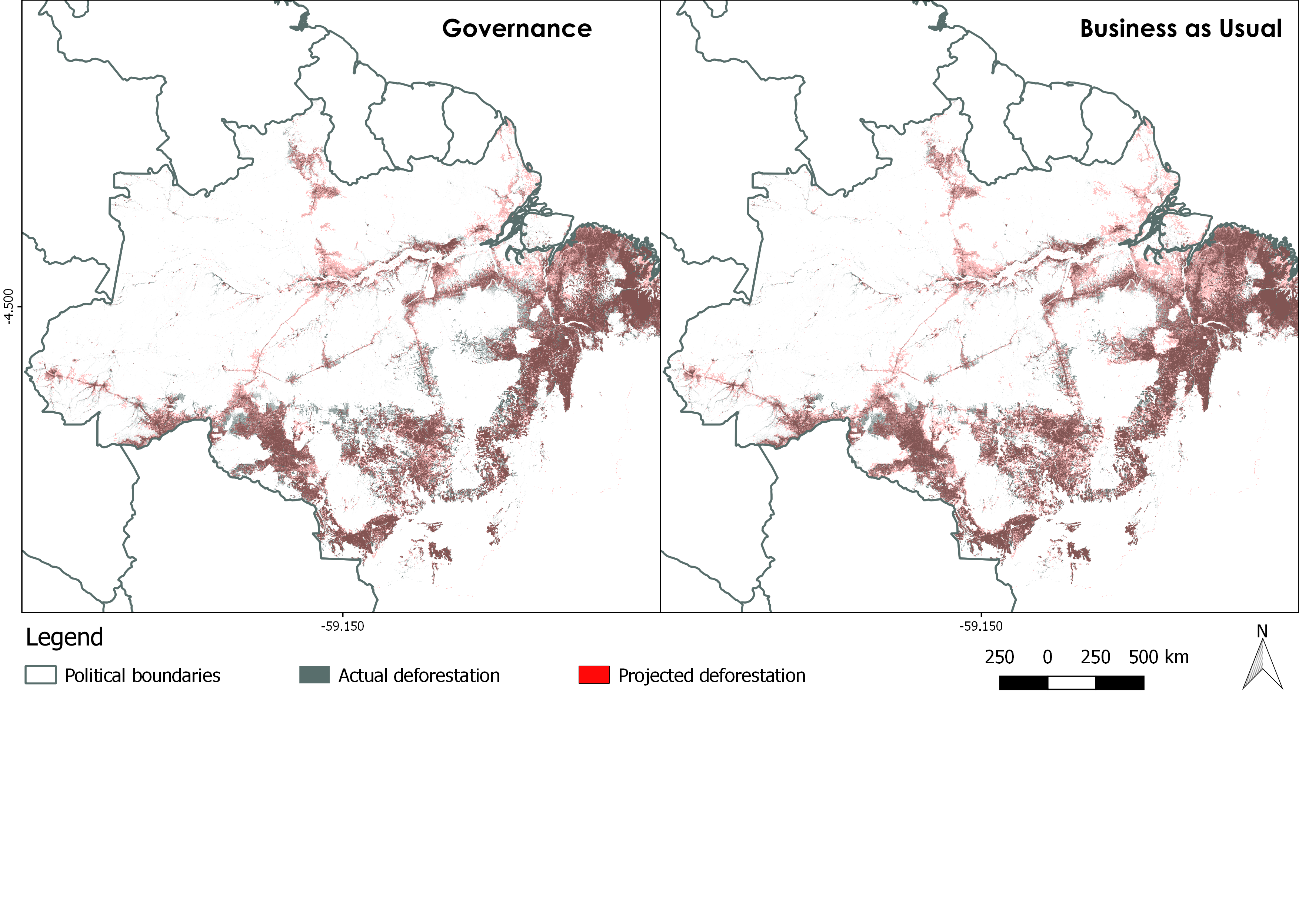


Figure S1. Map contrasting future deforestation projected by Soares-Filho et al. (2006) for the year 2018 and the accumulated deforestation mapped by PRODES (2019) for the same year for Brazil.


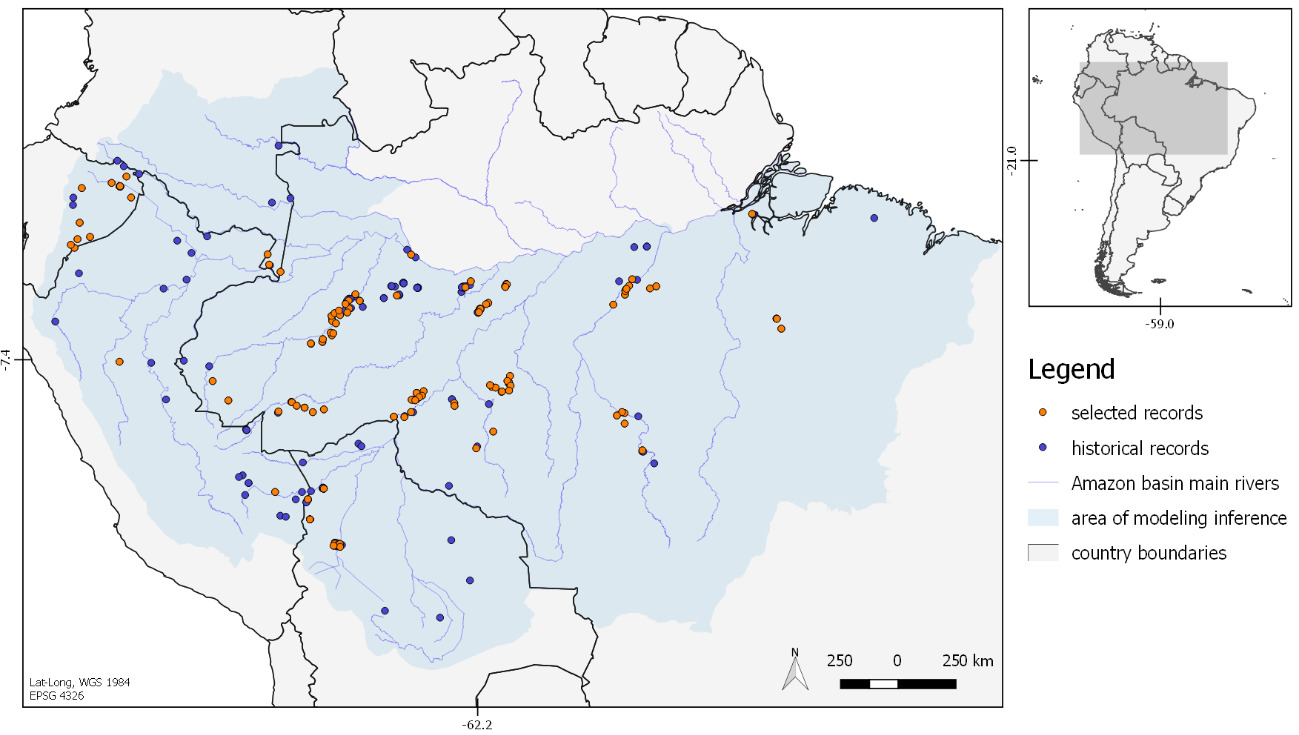


Figure S2. Short-eared dog (Atelocynus microtis) record locations. Historical records (n=307) are all published and unpublished records compiled by this study. Selected records (n=97) are those used in the species distribution modeling after controlling for spatial sampling bias and excluding records from before the year 2000. See Appendix 1 for full record details.


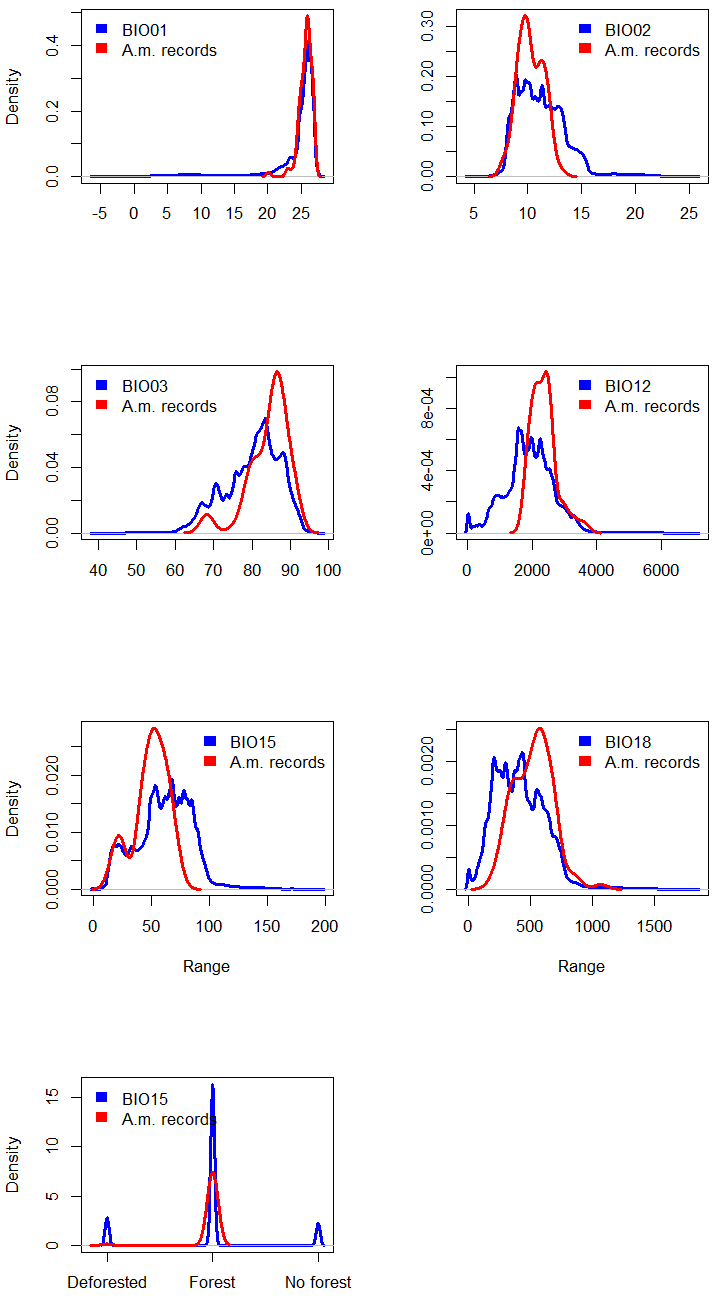


*Figure S3. Comparison of distributions of predictor variable values across our Maxent modeled area and values at the short-eared dog (*Atelocynus microtis*) record locations used for species distribution modeling.*


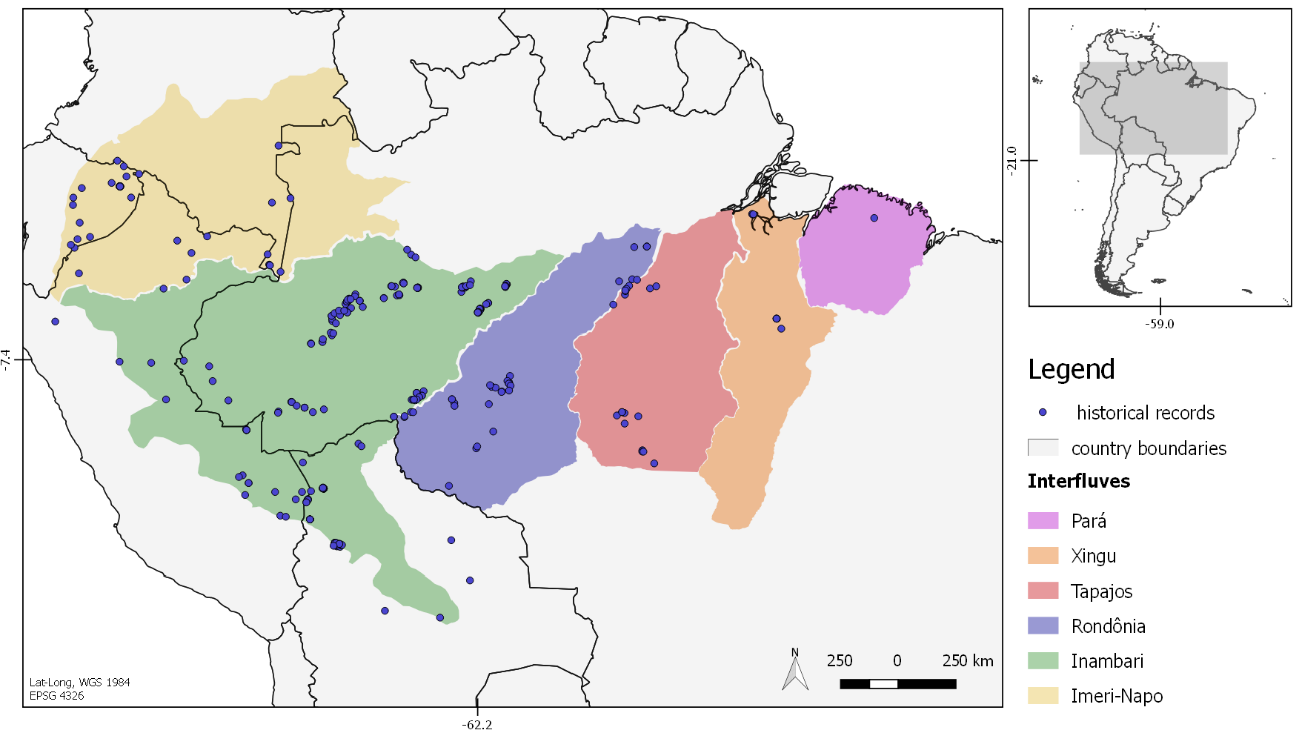


Figure S4. Amazon main interfluve regions (with modifications from Ribas et al. 2012) and short-eared dog (Atelocynus microtis) historical records.

Table S3. Predicted current distribution [km^2^] for the short-eared dog (Atelocynus microtis) and proportion of area reduction (%) by 2027 and 2045 under two alternative conservation scenarios, Governance and Business-as-Usual, for the Amazon basin (following Soares-Filho et al., 2006), based on Maxent model with land cover and climate covariates. Values were calculated within and outside protected areas (PA), as well as for each country with species records, and for different Amazon basin interfluves. Estimated area reductions in km^2^ (instead of proportions) are available in Table S4 and Table S5.

|  | **Current distribution area (km^2^)** | **Area reduction (%)** | | | |
| --- | --- | --- | --- | --- | --- |
|  |  | **Governance** | | **Business as Usual** | |
|  |  | **2027** | **2045** | **2027** | **2045** |
| Total | 4,302,532 | 13.8 | 14.7 | 17.7 | 21.2 |
| within PA | 1,882,654 | 7.4 | 7.7 | 9.5 | 13.0 |
| outside PA | 2,419,877 | 18.8 | 20.2 | 24.0 | 27.7 |
| **per country** |  |  |  |  |  |
| Colombia | 425,293 | 4.2 | 5.1 | 4.9 | 6.2 |
| Ecuador | 85,015 | 11.7 | 17.7 | 13.8 | 23.9 |
| Peru | 633,960 | 7.9 | 9.2 | 8.1 | 10.2 |
| Bolivia | 361,711 | 24.6 | 26.2 | 25.5 | 29.0 |
| Brazil | 2,793,245 | 15.2 | 15.8 | 20.9 | 24.9 |
| **per interfluve** |  |  |  |  |  |
| Pará | 77,769 | 34.0 | 34.1 | 38.2 | 38.3 |
| Xingu | 282,455 | 27.1 | 27.8 | 41.7 | 48.2 |
| Tapajós | 508,120 | 15.8 | 17.2 | 24.5 | 33.3 |
| Rondônia | 545,231 | 10.4 | 11.0 | 17.2 | 22.0 |
| Inambari | 1,349,937 | 5.0 | 5.3 | 5.0 | 5.7 |
| Imeri-Napo | 856,195 | 3.2 | 4.0 | 3.7 | 5.0 |

Table S4. Predicted area loss (km^2^) of the short-eared dog (Atelocynus microtis) distribution by 2027 and 2045 taking into account two alternative conservation scenarios, Governance and Business-as-Usual, for the Amazon basin (following Soares-Filho et al., 2006). Distribution areas were estimated with Maxent using land cover and climatic variables. Areas were calculated within and outside protected areas (PA), as well as for each country with species records, and for different Amazon basin interfluves.

|  | **Governance** | | **Business as Usual** | |
| --- | --- | --- | --- | --- |
|  | **2027** | **2045** | **2027** | **2045** |
| Total | 593,864 | 632,872 | 759,411 | 913,297 |
| within PA | 139,860 | 145,251 | 178,285 | 243,847 |
| outside PA | 454,004 | 487,621 | 581,126 | 669,451 |
| **per country** |  |  |  |  |
| Colombia | 17,983 | 21,611 | 20,766 | 26,190 |
| Ecuador | 9,918 | 15,038 | 11,731 | 20,306 |
| Peru | 50,019 | 58,630 | 51,169 | 64,559 |
| Bolivia | 89,051 | 94,598 | 92,242 | 104,918 |
| Brazil | 425,956 | 442,044 | 582,468 | 696,272 |
| **per interfluves** |  |  |  |  |
| Pará | 26,448 | 26,491 | 29,678 | 29,788 |
| Xingu | 76,536 | 78,471 | 117,711 | 136,234 |
| Tapajós | 80,153 | 87,472 | 124,721 | 169,140 |
| Rondônia | 56,477 | 59,861 | 94,042 | 120,088 |
| Inambari | 67,113 | 71,987 | 67,791 | 76,846 |
| Imeri-Napo | 27,315 | 33,970 | 31,459 | 42,834 |

Table S5. Proportion of area (%) of current short-eared dog (Atelocynus microtis) distribution expected to have a reduction of habitat suitability >50% by 2027 and 2045 under two conservation scenarios, Governance and Business-as-Usual, for the Amazon basin (following Soares-Filho et al., 2006). Distributions were estimated with Maxent using land cover and climatic variables. Values were calculated within and outside protected areas (PA), as well as for each country with species records, and for different Amazon basin interfluves. Estimated areas with suitability reduction in km^2^ (instead of proportions) are available in Table S7.

|  | **Habitat suitability reduction (%)** | | | |
| --- | --- | --- | --- | --- |
|  | **Governance** | | **Business as Usual** | |
|  | **2027** | **2045** | **2027** | **2045** |
| Total | 9.9 | 13.2 | 14.3 | 26.7 |
| within PA | 3.3 | 5.3 | 7.2 | 19.8 |
| outside PA | 15.8 | 20.3 | 20.8 | 33.2 |
| **per country** |  |  |  |  |
| Colombia | 8.5 | 11.7 | 8.7 | 13.7 |
| Ecuador | 2.3 | 3.1 | 2.5 | 4.7 |
| Peru | 3.9 | 5.2 | 4.5 | 7.0 |
| Bolivia | 12.9 | 15.6 | 14.1 | 19.5 |
| Brazil | 11.6 | 15.5 | 18.3 | 36.1 |
| **per interfluves** |  |  |  |  |
| Pará | 58.5 | 58.7 | 66.8 | 67.2 |
| Xingu | 11.3 | 11.5 | 29.0 | 39.5 |
| Tapajós | 9.3 | 12.2 | 21.6 | 49.4 |
| Rondônia | 13.7 | 20.5 | 19.0 | 45.3 |
| Inambari | 11.1 | 15.3 | 13.8 | 26.3 |
| Imeri-Napo | 3.7 | 5.2 | 3.9 | 6.3 |

Table S6. Predicted area (km^2^) within short-eared dog (Atelocynus microtis) distribution expected to have a reduction of habitat suitability >50% by 2027 and 2045 taking into account two alternative conservation scenarios, Governance and Business-as-Usual, for the Amazon basin (following Soares-Filho et al., 2006). Distribution areas were estimated with Maxent using land cover and climatic variables. Areas were calculated within and outside protected areas (PA), as well as for each country with species records, and for different Amazon basin interfluves.

|  | **Governance** | | **Business as Usual** | |
| --- | --- | --- | --- | --- |
|  | **2027** | **2045** | **2027** | **2045** |
| Total | 368,646 | 485,338 | 505,993 | 905,056 |
| within PA | 57,814 | 92,651 | 123,100 | 324,650 |
| outside PA | 310,832 | 392,687 | 382,893 | 580,406 |
| **per country** |  |  |  |  |
| Colombia | 34,509 | 47,242 | 35,094 | 54,600 |
| Ecuador | 1,760 | 2,159 | 1,842 | 3,066 |
| Peru | 22,664 | 29,942 | 26,320 | 39,692 |
| Bolivia | 35,204 | 41,695 | 37,864 | 49,956 |
| Brazil | 273,896 | 363,680 | 403,829 | 756,370 |
| **per interfluve** |  |  |  |  |
| Pará | 29,998 | 30,084 | 32,129 | 32,258 |
| Xingu | 23,338 | 23,487 | 47,840 | 57,689 |
| Tapajós | 39,613 | 51,156 | 82,851 | 167,557 |
| Rondônia | 66,727 | 99,646 | 85,546 | 192,777 |
| Inambari | 142,066 | 196,124 | 176,461 | 335,171 |
| Imeri-Napo | 30,623 | 42,812 | 32,051 | 51,315 |

Table S7. Predicted area (km^2^) of short-eared dog (Atelocynus microtis) distribution expected to be lost or have a reduction of habitat suitability >50% by 2027 and 2045 taking into account two alternative conservation scenarios, Governance and Business-as-Usual, for the Amazon basin (following Soares-Filho et al., 2006). Distribution areas were estimated with Maxent using land cover and climatic variables. Areas were calculated within and outside protected areas (PA), as well as for each country with species records, and for different Amazon basin interfluves.

|  | **Governance** | | **Business as Usual** | |
| --- | --- | --- | --- | --- |
|  | **2027** | **2045** | **2027** | **2045** |
| Total | 962,425 | 1,118,125 | 1,265,277 | 1,818,225 |
| within PA | 197,674 | 237,902 | 301,385 | 568,496 |
| outside PA | 764,750 | 880,223 | 963,892 | 1,249,729 |
| **per country** |  |  |  |  |
| Colombia | 52,492 | 68,853 | 55,860 | 80,790 |
| Ecuador | 11,678 | 17,197 | 13,573 | 23,372 |
| Peru | 72,684 | 88,573 | 77,489 | 104,251 |
| Bolivia | 124,254 | 136,293 | 130,106 | 154,874 |
| Brazil | 699,767 | 805,638 | 986,168 | 1,452,513 |
| **per interfluves** |  |  |  |  |
| Pará | 56,447 | 56,575 | 61,807 | 62,045 |
| Xingu | 99,873 | 101,958 | 165,551 | 193,923 |
| Tapajós | 119,766 | 138,628 | 207,572 | 336,697 |
| Rondônia | 123,119 | 159,422 | 179,460 | 312,738 |
| Inambari | 209,179 | 268,111 | 244,252 | 412,017 |
| Imeri-Napo | 57,938 | 76,782 | 63,510 | 94,149 |

Table S8. Results of AIC-based model selection for occupancy models for short-eared dog (Atelocynus microtis) in the Southern Brazilian Amazon. Single-covariate single-season occupancy models were compared to select the best buffer radius around each camera-trap for each covariate. The site covariates tested were forest cover (FOR), forest edge density (ED), forest patch density (PD), distance to water (Water), distance to roads (Road) elevation (Elev) and Human Footprint Index) (HFI) in different buffers (radius of 0.5, 1, 1.5, 3, 5, 10 km). Effort (EFF) was a survey covariate included in all models. ΔAIC is the relative difference in AIC values compared with the top ranked model.

| **Model** | | **Parameters** | | **AIC** | | | Δ**AIC** | |
| --- | --- | --- | --- | --- | --- | --- | --- | --- |
| **Forest cover** | |  | |  | | |  | |
| p(EFF) psi(FOR0.5) | | 4 | | 178.7 | | | 0 | |
| p(EFF) psi(FOR1) | | 4 | | 179.9 | | | 1.20 | |
| p(EFF) psi(FOR1.5) | | 4 | | 182.4 | | | 3.68 | |
| p(EFF) psi(FOR3) | | 4 | | 183.3 | | | 4.61 | |
| p(EFF) psi(FOR5) | | 4 | | 185.0 | | | 6.32 | |
| p(EFF) psi(.) | | 3 | | 186.2 | | | 7.50 | |
| p(EFF) psi(FOR10) | | 4 | | 186.5 | | | 7.80 | |
| **Edge density** | |  | |  | | |  | |
| p(EFF) psi(ED1) | | 4 | | 184.8 | | | 0 | |
| p(EFF) psi(ED3) | | 4 | | 185.4 | | | 0.62 | |
| p(EFF) psi(ED1.5) | | 4 | | 185.6 | | | 0.77 | |
| p(EFF) psi(ED10) | | 4 | | 186.2 | | | 1.38 | |
| p(EFF) psi(.) | | 3 | | 186.2 | | | 1.38 | |
| p(EFF) psi(ED5) | | 4 | | 186.7 | | | 1.86 | |
| p(EFF) psi(ED0.5) | | 4 | | 188.2 | | | 3.38 | |
| **Patch density** | |  | |  | | |  | |
| p(EFF) psi(.) | | 3 | | 186.2 | | | 0 | |
| p(EFF) psi(PD1.5) | | 4 | | 186.8 | | | 0.58 | |
| p(EFF) psi(PD1) | | 4 | | 187.2 | | | 0.99 | |
| p(EFF) psi(PD3) | | 4 | | 187.6 | | | 1.44 | |
| p(EFF) psi(PD10) | | 4 | | 187.8 | | | 1.63 | |
| p(EFF) psi(PD5) | | 4 | | 188.1 | | | 1.95 | |
| p(EFF) psi(PD0.5) | | 4 | | 188.2 | | | 1.97 | |
| **Distance to water^1^** |  | |  | | |  | |  |
| p(EFF) psi(.) | 3 | | | | 186.2 | | 0 | |
| p(EFF) psi(Water_b5) | 4 | | | | 187.5 | | 1.25 | |
| p(EFF) psi(Water_b10) | 4 | | | | 187.6 | | 1.36 | |
| p(EFF) psi(Water_b3) | 4 | | | | 187.8 | | 1.58 | |
| p(EFF) psi(Water_b1.5) | 4 | | | | 188.2 | | 1.92 | |
| p(EFF) psi(Water_b1) | 4 | | | | 188.2 | | 1.96 | |
| p(EFF) psi(Water_b0.5 | 4 | | | | 188.2 | | 1.99 | |
| **Distance to roads^2^** |  | | | |  | |  | |
| p(EFF) psi(.) | 3 | | | | 186.2 | | 0 | |
| p(EFF) psi(Road_b5) | 4 | | | | 195.4 | | 9.16 | |
| p(EFF) psi(Road_b3) | 4 | | | | 195.5 | | 9.3 | |
| p(EFF) psi(Road_b1.5) | 4 | | | | 196.5 | | 10.3 | |
| p(EFF) psi(Road_b1) | 4 | | | | 196.7 | | 10.41 | |
| p(EFF) psi(Road_b0.5) | 4 | | | | 196.7 | | 10.45 | |
| p(EFF) psi(Road_b10) | 4 | | | | 229.9 | | 43.65 | |
| **Elevation^3^** |  | | | |  | |  | |
| p(EFF) psi(.) | 3 | | | | 186.2 | | 0 | |
| p(EFF) psi(Elev_b1.5) | 4 | | | | 187.7 | | 1.41 | |
| p(EFF) psi(Elev_b3) | 4 | | | | 187.7 | | 1.42 | |
| p(EFF) psi(Elev_b1) | 4 | | | | 187.8 | | 1.61 | |
| p(EFF) psi(Elev_b0.5) | 4 | | | | 187.9 | | 1.71 | |
| p(EFF) psi(Elev_b5) | 4 | | | | 188.0 | | 1.8 | |
| p(EFF) psi(Elev_b10) | 4 | | | | 188.2 | | 1.95 | |
| **Human Footprint Index^4^** |  | | | |  | |  | |
| p(EFF) psi(.) | 3 | | | | 186.2 | | 0 | |
| p(EFF) psi(HFI_b0.5) | 4 | | | | 186.5 | | 0.28 | |
| p(EFF) psi(HFI_b1) | 4 | | | | 187.0 | | 0.74 | |
| p(EFF) psi(HFI_b1.5) | 4 | | | | 187.4 | | 1.14 | |
| p(EFF) psi(HFI_b3) | 4 | | | | 187.9 | | 1.61 | |
| p(EFF) psi(HFI_b10) | 4 | | | | 187.9 | | 1.63 | |
| p(EFF) psi(HFI_b5) | 4 | | | | 188.0 | | 1.77 | |

**^1^** data source: Seyler et al. (2009)

**^2^** data source: RAISG, available at <https://www.amazoniasocioambiental.org/en/maps/#download>, assessed on 30 Sept. 2019.

**^3^** data source: Jarvis et al. (2008)

**^4^** data source: Venter et al. (2016)

Table 9. Parameter estimates (with standard errors and 95% confidence intervals) from the best occupancy model (p(EFF) psi(FOR0.5+ ED1)) for short-eared dogs (Atelocynus microtis) in the Southern Brazilian Amazon.

| **Covariate** | **Estimate** | **SE** | **Lower CI** | **Upper CI** |
| --- | --- | --- | --- | --- |
| **Occupancy** |  |  |  |  |
| Intercept | -3.795 | 3.196 | -1.005 | 2.468 |
| FOR0.5 | 0.036 | 0.019 | -0.001 | 0.073 |
| ED1 | -0.017 | 0.068 | -0.150 | 0.116 |
| **Detection** |  |  |  |  |
| Intercept | -1.443 | 1.341 | -4.073 | 1.186 |
| EFF | 0.024 | 0.067 | -0.108 | 0.156 |


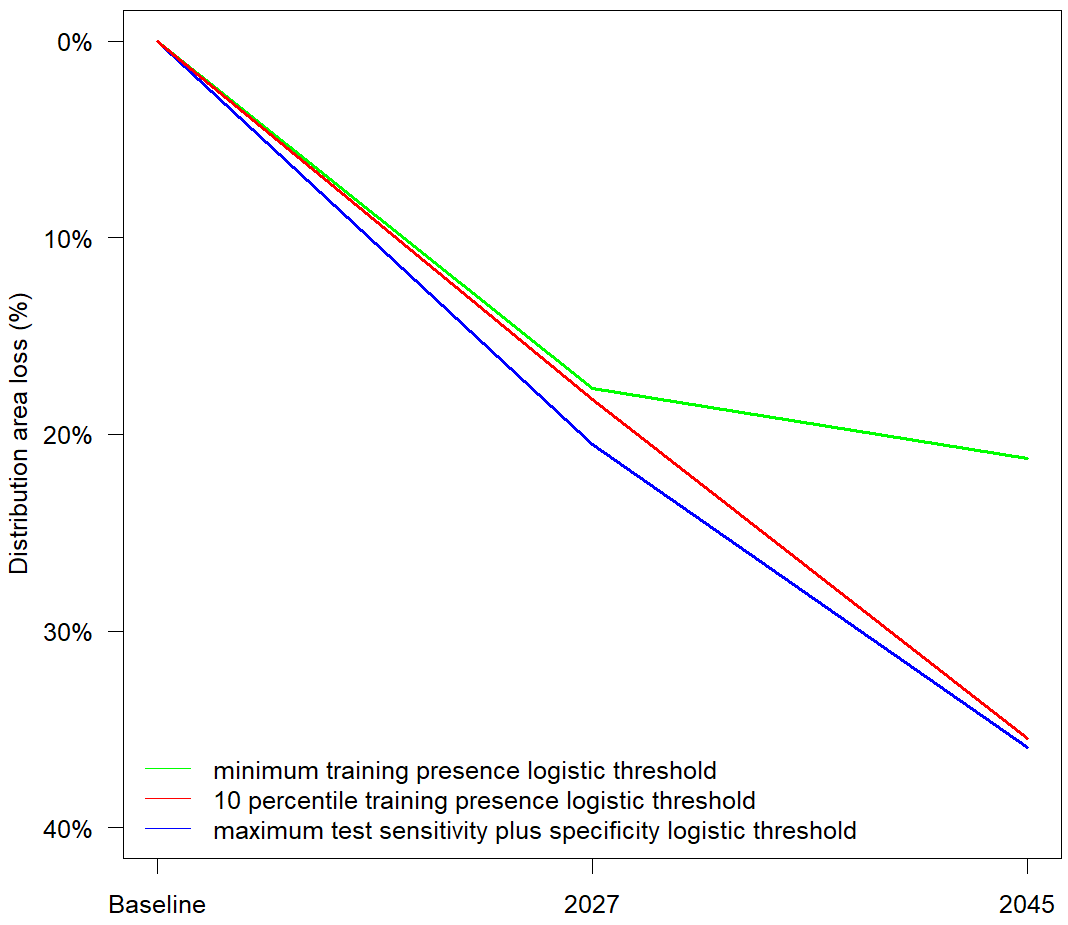


Figure S5. Proportion of the short-eared dog (Atelocynus microtis) distribution area expected to be lost by 2027 and 2045 according to the Business-as-Usual conservation scenarios modeled by Soares-Filho (2006), using three different threshold of habitat suitability. Distributions areas were estimated with Maxent using land cover and climatic variables. In this study, the minimum training presence logistic threshold was used (see Methods section for details on threshold selection).

Table S10. Tree cover area, tree cover area loss and tree cover area loss as percent of year 2000 tree cover by country (Hansen et al., 2013), for all contries with confirmed records of the short-eared dog (Atelocynus microtis). Tree covered pixels are those that had at least 75% of their area covered by vegetation taller than 5m in height.

| **Country** | **Tree cover in 2000 in km^2^** | **Tree cover loss between 2001-2016 in Km^2^** | **Tree cover loss between 2001-2016 in %** |
| --- | --- | --- | --- |
| Bolivia | 478,379 | 31,424 | 6.57 |
| Brazil | 4,292,337 | 359,121 | 8.37 |
| Colombia | 721,707 | 29,577 | 4.10 |
| Ecuador | 158,777 | 6,092 | 3.84 |
| Peru | 75,472 | 22,985 | 30.46 |

**References**

Hansen, M.C., Potapov, P. V., Moore, R., Hancher, M., Turubanova, S.A., Tyukavina, A., Thau, D., Stehman, S. V., Goetz, S.J., Loveland, T.R., Kommareddy, A., Egorov, A., Chini, L., Justice, C.O., Townshend, J.R.G., 2013. Hansen/UMD/Google/USGS/NASA Tree Cover Loss and Gain Area [WWW Document]. Univ. Maryland, Google, USGS, NASA. URL www.globalforestwatch.org (accessed 5.16.18).

Jarvis, A., Reuter, H.I., Nelson, A., Guevara, E., 2008. Hole-filled SRTM for the globe Version 4. available from CGIAR-CSI SRTM 90m Database (http//srtm. csi. cgiar. org) 15.

Seyler, F., Muller, F., Cochonneau, G., Guimarães, L., Guyot, J.L., 2009. Watershed delineation for the Amazon sub‐basin system using GTOPO30 DEM and a drainage network extracted from JERS SAR images. Hydrol. Process. An Int. J. 23, 3173–3185.

Soares-Filho, B.S., Nepstad, D.C., Curran, L.M., Cerqueira, G.C., Garcia, R.A., Ramos, C.A., Voll, E., McDonald, A., Lefebvre, P., Schlesinger, P., 2006. Modelling conservation in the Amazon basin. Nature 440, 520–523.

Venter, O., Sanderson, E.W., Magrach, A., Allan, J.R., Beher, J., Jones, K.R., Possingham, H.P., Laurance, W.F., Wood, P., Fekete, B.M., 2016. Global terrestrial Human Footprint maps for 1993 and 2009. Sci. data 3, 160067.
